# Intention to use and perceived satisfaction with a digital tool for dementia prevention among the Dutch general public: a cross-sectional study

**DOI:** 10.1186/s12889-025-23777-y

**Published:** 2025-09-30

**Authors:** Tanja J. de Rijke, Leonie N.C. Visser, Kyra K.M. Kaijser, Jeroen Bruinsma, Susan J. Oudbier, Irene Heger, Wiesje M. van der Flier, Ellen M.A. Smets, Thomas Engelsma

**Affiliations:** 1 Department of Medical Psychology, Amsterdam UMC location University of Amsterdam, Meibergdreef 9, Amsterdam, AZ 1105, the Netherlands; 2 Amsterdam Public Health Research Institute, Personalised Medicine, Amsterdam, The Netherlands; 3 Alzheimer Center Amsterdam, Department of Neurology, Amsterdam UMC, Vrije Universiteit Amsterdam, Amsterdam, The Netherlands; 4 Amsterdam Neuroscience, Amsterdam, The Netherlands; 5 Amsterdam Public Health Research Institute, Digital Health, Amsterdam, The Netherlands; 6 Division of Clinical Geriatrics, Center for Alzheimer Research, Department of Neurobiology, Care Sciences and Society, Karolinska Institutet, Stockholm, Sweden; 7 Department of Bioethics & Medical Humanities, UMC Utrecht, Utrecht, The Netherlands; 8 Department of Health Promotion, Care and Public Health Research Institute (CAPHRI), Maastricht University, Maastricht, The Netherlands; 9 Outpatients Department, Amsterdam UMC, ‘Samen Digitaal’, Amsterdam, The Netherlands; 10 Department of Psychiatry and Neuropsychology, Mental Health and Neuroscience Research Institute (MHeNs), Maastricht University, Maastricht, The Netherlands; 11 Department of Epidemiology and Biostatistics, Amsterdam Neuroscience, VU University Medical Center, Amsterdam, The Netherlands; 12 Amsterdam Public Health Research Institute, Quality of Care, Amsterdam, The Netherlands; 13 Department of Medical Informatics, eHealth Living & Learning Lab Amsterdam, Amsterdam UMC location University of Amsterdam, Amsterdam, The Netherlands

**Keywords:** Alzheimer’s disease, Dementia, Subjective cognitive decline, Cognitively unimpaired, Public health, Digital health, EHealth, Accessibility, Equity, Digital divide

## Abstract

**Background:**

The increasing prevalence of dementia coincides with a shrinking healthcare workforce, highlighting the importance of dementia prevention. Digital health tools are smart devices and connected equipment that improve health and can be used by individuals themselves, such as apps, platforms, or wearables. These tools can be used to empower people in the general population to take care of their brain health and dementia prevention. We aimed to exploratively investigate the intention to use and perceived satisfaction with MijnBreincoach, a Dutch digital tool for dementia prevention among the general public, and their associated variables.

**Methods:**

For this observational, cross-sectional study, data was gathered via convenience and purposive sampling using a questionnaire among cognitively unimpaired people and people with self-reported cognitive complaints. The questionnaire covered variables, including gender, age, employment situation, educational attainment, perceived financial scarcity, health literacy, dementia risk, motivation for dementia prevention-related behaviour change, digital proficiency, digital acceptability, and technology adoption. MijnBreincoach was included as a use case to assess the outcome variables ‘intention to use’ and ‘perceived satisfaction’. Analyses included exploratory descriptives, correlations, and robust backward stepwise regression using Generalized Linear Models with 5-fold cross-validation.

**Results:**

Study participants (*n* = 673; mean age = 60.6 ± 13.9; female = 68%; education (low = 26.1%; medium = 35%; high = 38.9%)) had a mean intention to use of 3.1 ± 3 (min = 1; max = 5) and mean perceived satisfaction of 6.4 ± 2.4 (min = 0; max = 10). Educational attainment, digital proficiency, digital acceptability, technology adoption, and cues to action explain almost half (44%) of the variance in intention to use. For perceived satisfaction, 28.2% of the variance was explained by digital proficiency and technology adoption.

**Conclusion:**

In general, the Dutch general public has a moderate intention to use and perceived satisfaction with a digital tool for dementia prevention. Yet, large variation in scores was observed, which provides opportunity to tailor support for the use of these digital tools to different target groups.

**Supplementary Information:**

The online version contains supplementary material available at 10.1186/s12889-025-23777-y.

## Introduction

Dementia is a major public health problem [1]. The number of people with dementia worldwide is predicted to increase from 50 to 152 million by 2050 due to ageing populations and the existing lack of widely accessible effective treatment options [2]. This increasing prevalence of dementia coincides with a shrinking healthcare workforce, making it even harder to meet the demand for dementia-related healthcare in the future [3]. The most recent Lancet Commission on dementia prevention, intervention and care estimated that addressing modifiable risk factors could potentially prevent or delay approximately 45% of global dementia cases, highlighting the importance to be ambitious about dementia prevention [4]. Concurrently, we observe a more widespread trend within the healthcare sector with increased personal responsibility for one’s own health, including brain health in the context of dementia prevention [5, 6]. 

Digital health tools are increasingly available and have the potential to enable people in the general public to become actively involved in their own brain health [7, 8]. Digital health tools are *‘smart devices and connected equipment that improve health’* that can be used by individuals themselves, such as mobile apps, digital platforms, or wearables [9]. These type of tools are considered to be appropriate, accessible, affordable, sustainable, and have the potential to be highly scalable [9–11]. Additionally, digital health tools may allow users to access their own health information, which may improve their understanding of their condition, provides them control and accountability, and enables them to make well-informed decisions, all of which may empower individuals [12, 13]. Examples of digital health tools that were developed for the general public and/or patients include mobile or wearable technology to assess, detect, and monitor early-disease symptoms [14]. In the context of dementia prevention, the Dutch app MijnBreincoach is an example of a digital tool that provides insight into someone’s personal dementia risk profile, identifies room-for-improvement based on lifestyle, and provides support to make small steps towards improved brain health and dementia risk reduction [15, 16]. 

Regardless of the increasing availability of digital health tools and their potential benefits, adoption and sustainable use remain low, especially among people from diverse socioeconomic, ethnic, and linguistic backgrounds [17–19]. Previous research shows that people with diverse backgrounds are interested in using digital health tools [19], but may experience barriers due to digital and health literacy. Some scholars argue that we face a ‘digital divide’ due to long-lasting systemic disparities, in which digital use patterns differ by, for example, socioeconomic position [20]. Furthermore, a multitude of risk factors for dementia are documented as being more prevalent among minority groups, for instance, individuals with a lower socioeconomic position or with less educational attainment [21, 22]. However, others argue that digital tools may be more acceptable and easier to use for people with diverse backgrounds. For instance, people with low health literacy levels have been found to consider mHealth apps more acceptable and easier to use than other health promotion materials and screening questionnaires [23]. It is therefore important to investigate factors related to intention to use and satisfaction with digital health tools for dementia prevention among a diverse population to avoid potentially enlarging existing health disparities [24, 25]. 

Besides sociodemographic factors like gender and age, multiple other factors have been identified that could influence the intention to use a digital health tool, such as motivation or specific digital skills [26–33]. However, so far no research has systematically investigated which factors drive people’s intention to use and satisfaction. Thus, the aim of this study was to explore intention to use and perceived satisfaction with a digital tool for dementia prevention among people in the Dutch general public, and the associated variables of those outcomes.

## Methods

### Study design and recruitment

An observational, cross-sectional questionnaire study was conducted among people in the Dutch general public without a diagnosis of dementia, Alzheimer’s disease, or mild cognitive impairment. People who perceive a decline in memory or cognition without objective impairment(s) were also eligible to participate. Recruitment took place in two waves with the rationale to further enrich the study sample’s diversity in the second wave. Both waves included pilot testing of the questionnaire (see below). Data from wave I and wave II were merged into one data set prior to analyses (see supplements S2 for details).

Wave I was conducted in Spring 2023 using convenience sampling via online participant recruitment via the platform ‘Hersenonderzoek.nl’. This Dutch Brain Research Registry is an online register which facilitates pre-screening and recruitment of research participants for neuroscientific research [34]. The registry requires having an email address during registration for the registry. The entire adult population in the Netherlands was able to participate in wave I: they only were excluded in case of self-reported dementia, Alzheimer’s disease, or mild cognitive impairment. We included people aged 18 years or older, as young adults can already be exposed to dementia risk factors, such as smoking, air pollution, loud noise with unsafe listening practices, or excessive alcohol intake [35], in part due to increased experimentation and risk-taking behaviours (e.g. smoking, alcohol intake, or drug use) during young adulthood [35–38]. Since health behaviours that are established during young adulthood may have long-term health effects, addressing these at a young age highlights a window of opportunity for dementia risk reduction [35, 39, 40]. To maximize study sample diversity in our initial sample in wave I, people with lower and medium educational attainment were oversampled. Eligible participants were invited for study participation via e-mail. Participants who expressed interest in participating in this study received a link to a brief description of the study content, an elaborate information letter on the purpose and procedures, and a personalized link to the online version of the questionnaire. The questionnaire was developed and distributed using Survalyzer software [41]. Restricted access to the questionnaire was build-in to prevent individuals from participating multiple times.

Wave II aimed to further enrich study sample diversity in terms of digital proficiency, ethnicity, and socioeconomic position, by adopting purposive sampling with a variety of recruitment strategies from March to July 2024. The entire population in the Netherlands aged 40 years or older with no self-reported dementia, Alzheimer’s disease, or mild cognitive impairment was able to participate. For wave II, the focus was on individuals aged 40 years and over, as opposed to those aged 18 years and over. The objective was to enhance the sample of people in midlife (40–60 years) and beyond, as risk factors begin to accumulate and exert an influence from the age of 40 [42–45]. A separate manuscript reporting on the exact recruitment process and evaluation of the recruitment strategies is in preparation (see Kaijser et al., in preparation). The recruitment strategies comprised offline passive recruitment (e.g. radio advertisement and advertisement in public transport), online passive recruitment (e.g. recruitment via WhatsApp and Facebook groups), active in-person recruitment (e.g. recruitment in community centres and at community events), and active recruitment via champion organisations (e.g. elderly care professionals asking their clients to participate). People could indicate their interest to participate in-person, via e-mail, a phone call, text message, or WhatsApp. In wave II, it was possible to participate without having an email address. Both online and paper versions of the questionnaire were distributed, i.e., those recruited via offline methods could also opt to complete the questionnaire online and vice versa.

### Ethics

The Medical Ethics Committee of the Amsterdam UMC, location AMC, approved the study (W22_426 # 22.504). Participants were given a random identification number and data is stored in a controlled access storage in the Amsterdam UMC, location AMC. No one outside of the direct study team (TR, LV, ES, KK, TE) has access to these files. All participants provided informed consent prior to participation. The study complies with ethical standards of the Helsinki Declaration [46]. For reporting of the methods and results, we used the Checklist for Reporting of Survey Studies (CROSS) [47]. 

### Variables and instruments

**Table 1:** Overview of variables and instruments

Variables	Instruments	Scoring	Cronbach’s alpha based on our sample
Outcome variables			
Use case assessing behavioral intentions and perception of usability			
Intention to use	Subscale of the Unified Theory of Acceptance & Use of Technology 2 (UTAUT-2) [48] measuring technology adoption	A three-item five-point Likert scale is used with 1 being ‘strongly agree’ and 5 being ‘strongly disagree’. For analysis purposes, the Likert scale was recoded into 1 being ‘strongly disagree’ and 5 being ‘strongly agree’, so that higher total scores indicate a higher intention (min. 1; max. 5).	α = 0.700
Perceived satisfaction	Net Promoter Score (NPS) [49]	The NPS is a one-item question measured on a scale of 0 being ‘not at all likely’ and 10 being ‘very likely’.	-
***Associated variables***			
Sociodemographics			
Gender	-	*	-
Age in years	-	*	-
Employment situation	Self-developed item	*	-
Educational attainment	Dutch CBS division by Pleijers & De Vries [50].	1-item scale from 1 (low educational attainment) to 10 (high educational attainment) based on the Dutch educational system. Classification was done as follows: low (none or elementary school, primary or preparatory vocational education (vso, vmbo-b, vmbo-k, vmbo-g, vmbo-t, mavo, mulo, lts, leao, lhno of meao), lower grades of secondary school of HAVO or VWO, or assistant training (MBO-1)), medium (higher grades of secondary school of HAVO, VWO, HBS, or MMS, basic vocational training (MBO-2), vocational training (MBO-3), and middle management and specialist education (MBO-4)), and high (higher vocational education (HBO, HEAO, or HTS) and university).	-
Perceived financial scarcity	Psychological Inventory of Financial Scarcity (PIFS-4)[51, 52]	4-item scale in which answers are given on a seven-point Likert scale ranging from strongly disagree (1 point) to strongly agree (7 points), so higher scores (min. 4; max. 28) reflect greater perceived experience financial scarcity [51].	α = 0.796
(Perceived) dementia risk and motivation for behaviour change			
Health literacy	Functional scale of the Functional, Communicative, and Critical Health Literacy (FCCHL) scale [53, 54]	A four-item four-point Likert scale ranging from 1 (never) to four (often), so higher scores (min. 1; max. 4) reflect lower health literacy.	α = 0.700
Dementia risk	Lifestyle for Brain Health scores (LIBRA) [55]	Twelve items measuring LIBRA scores. Total scores range from − 5.9 (min.) to 12.7 (max.), with a score above 1 being associated with higher scores reflecting a higher risk of dementia (and lower scores reflecting better brain health) [56, 57].	-
Motivation for dementia prevention-related behavior change*	Shortened version of the Motivation to Change Lifestyle and Health Behaviours for Dementia Risk Reduction scale (MCLHB-DRR) [58, 59].	Fourteen-items, divided over seven subscales, answered on a five-point Likert scale. For analysis purposes, the Likert scale was recoded into 1 (low motivation) to 5 (strong motivation)(min. 1; max. 5).	Perceived susceptibility: N/A**; perceived severity: α = 0.659; perceived benefits: α = 0.390; perceived barriers: α = 0.673; cues to action: α = 0.692; general health motivation: N/A**; self-efficacy: N/A**
Digital proficiency and acceptability			
Digital proficiency	Mobile Device Proficiency Questionnaire (MDPQ-16) [60]	A sixteen-item five-point Likert scale is used with 1 being ‘never tried’ and 5 being ‘very easy’, so that higher scores reflect a higher mobile device proficiency (min. 8; max. 40).	α = 0.927
Digital acceptability	Trust, perceived risk, and resistance to change items of mHealth Technology Acceptance Model (MoHTAM) [61]	Six questions are asked using a five-point Likert scale, with 1 being ‘completely agree’ and 5 being ‘strongly disagree’. A lower score means lower trust, perceived risk, and acceptance of technology (min. 6; max. 30).	-
Use case assessing behavioral intentions and perception of usability			
Technology adoption**	Unified Theory of Acceptance & Use of Technology 2 (UTAUT-2) [48]	A fourteen-item five-point Likert scale is used with 1 being ‘strongly agree’ and 5 being ‘strongly disagree’. For analysis purposes, the Likert scale was recoded into 1 being ‘strongly disagree’ and 5 being ‘strongly agree’, so that higher total scores indicate a higher intention (min. 1; max. 5).	α = 0.883

*For exact classification, see published metadata via 10.34894/Z74Q0Y

**excluding ‘intention to use’ items

**N/A, because subscale consists of one item

The questionnaire was developed for this study and informed by factors as described by the I-Change Model and the Causal and Sequential Model of Digital Technology Access (see supplements S1 for an elaborate explanation) [26, 27]. The questionnaire comprised the following parts: (1) sociodemographics, (2) (perceived) dementia risk and motivation for behaviour change, (3) digital proficiency and acceptability, and (4) a use case assessing technology adoption, intention to use, and perceived satisfaction (see Table 1). Intention to use (measured via the subscale ‘intention to use’ from the Unified Theory of Acceptance and Use of Technology (UTAUT-2) [48]) and perceived satisfaction (measured via the Net Promoter Score (NPS) [49]) were the outcome measures in this study. A full version of the final questionnaire is published previously in the online repository Dataverse (see: 10.34894/Z74Q0Y).

#### Sociodemographic variables

To assess *sociodemographic variables*, we asked participants to fill in their gender, age in years, employment situation, and educational attainment (see Table 1). *Financial scarcity* was measured using the PIFS-4, which assesses perceptions of insufficient financial resources, lack of control over someone’s financial situation, responses to financial rumination and worry, and a short-term focus [51, 52]. 

#### (Perceived) dementia risk and motivation for behaviour change

*Health literacy* was measured via the functional scale of the FCCHL, which measures someone’s ability to read and understand health-related information [53, 54]. The wording of the original Dutch version was adjusted slightly, due to item comprehension issues in the pilot tests [54]. *Dementia risk* was assessed using the LIBRA index, which measures someone’s potential for dementia prevention by quantifying twelve modifiable risk and protective factors for dementia (e.g. physical inactivity, hypertension, and cognitive activity) [55]. *Motivation for dementia prevention-related behaviour change* was assessed using a Dutch shortened version of the MCLHB-DRR with seven subscales on perceived susceptibility, perceived severity, perceived benefits, perceived barriers, cues to action, general health motivation, and self-efficacy [58]. 

#### Digital proficiency and digital acceptability

*Digital proficiency* was assessed using the MDPQ-16, which measures basic and advanced mobile device proficiency comprising nine subscales on mobile device basics, communication, data and file storage, internet, calendar, entertainment, privacy, troubleshooting, and software management [60]. *Digital acceptability* was assessed using items on trust, perceived risk, and resistance to change from the MoHTAM [61]. 

#### Use case assessing intention to use and perceptions of usability

A use case was presented in the questionnaire to provide participants with a clear picture of a digital tool and engage with a hypothetical scenario. The data described in this manuscript relate to the use case of ‘MijnBreincoach’ (https://www.mijnbreincoach.eu/) [15, 16]. MijnBreincoach was selected as a use case because it is in Dutch, is accessible on multiple devices, and is at a mid-maturity stage in its development [62]. A mid-maturity demonstration phase assesses the costs and implementation requirements, in which the effect of the tool is tested in an uncontrolled situation limited to a certain population or geography [62]. The use case started with a short description of the context, followed by a brief explanation, and screenshots of MijnBreincoach (see metadata for questionnaire containing the use case; 10.34894/Z74Q0Y). This use case assessed technology adoption and the outcome variables intention to use and perceived satisfaction [48, 63]. For this study, the subscale price value of the model used to measure technology adoption was not included as end-users do not bear the costs for using MijnBreincoach.

### Pilot testing

For wave I, the questionnaire was pilot tested among three persons who met the inclusion criteria (33.3% female; mean age 71) via a short interview after completion to check for clarity, sentence formulation, and completion time. Pilot testing led to some changes for clarification purposes and these data were not taken into account for analyses. Pilot participants of wave I could provide feedback via written comments in a designated box and via a short interview after completion. Pilot testers as well as participants from wave I indicated that the questionnaire was lengthy, and we therefore shortened the questionnaire by removing items or sections of items based on data and discussions with the team (see supplements S2 and metadata for elaborate information; 10.34894/Z74Q0Y). The shortened version of the questionnaire was pilot tested prior to use in wave II via a short interview after completion among 18 participants (33.3% female; mean age 57.6 years) and the language was checked by Stichting ABC, which is a Dutch organization specializing in literacy with the goal to improve readability and accessibility. The pilot tests and language check led to some final textual changes.

### Data processing and analyses

Analyses were conducted using IBM SPSS version 28, with α = 0.05 set as the threshold for significance [64]. Missing data points, including those arising from differences in questions between wave I and wave II, were excluded from analysis for that specific item. Exploratory demographic and correlation analyses were conducted using Pearson’s correlation or Spearman’s rho (see supplements S4 for correlation tables). Data regarding the eleven potential associated variables (as described above) were collected in addition to the outcome variables intention to use and perceived satisfaction. As the total score of the MCLHB-DRR is not informative in general, subscales were included in the analysis if they demonstrated a significant correlation with an associated variable. We performed a robust covariance matrix estimator for backward stepwise regression using generalized linear models (GLM). Backward stepwise regression via GLM avoids the suppressor effect [65], and captures potentially important interactions by starting with all possible associated variables (see Table 1) [66], which may yield more stable results compared to forward regression. For the GLM analysis, all factors and covariates were included in a single model. The model was then iteratively refined by removing the factor or covariate with the highest p-value until all remaining factors and covariates were statistically significant (α = 0.05). For *p*-values, we used the sum of squares (Type III) output, since the results hereof do not depend on order within the model [67]. After running the first model fit, we checked for heteroscedasticity and normality of errors of the residuals of intention to use and perceived satisfaction. Based on visual inspection of the scatter plots and histograms of the residuals, we decided to perform a robust regression via a robust estimator covariance matrix. We also performed 5-fold cross-validation to evaluate model stability and predictive power [68]. This approach helped to assess the performance of the model. We used 5-fold cross-validation to provide an honest estimate of out-of-sample performance on associated data to avoid optimism due to overfitting in our evaluation. To evaluate model performance, the data was randomly split five times into balanced training (80%) and testing sets (20%). We averaged the five R-squared change values of the testing data set (*n* = 20%) resulting in a mean R-squared change.

Variables were considered robust if they appeared in the baseline model all five times during 5-fold cross-validation. Finally, we performed subgroup analyses by assessing whether there were any significant differences between age groups (< 40 group versus ≥ 40 group; <60 versus ≥ 60 group) using a t-test on mean scores of intention to use and perceived satisfaction, and running backward stepwise regression using GLM per age group when significant. The cut-off points of 40 and 60 were chosen, since midlife and older age are considered pivotal periods for dementia prevention [69]. 

## Results

For wave I, 64.7% of invited participants completed the questionnaire compared to 43.8% in wave II. The merged dataset of wave I and wave II consisted of *n* = 673 participants. Study participants were on average 60.6 ± 13.9 years, female (68%), and had varying educational attainment (low = 26.1%; medium = 35%; high = 38.9%)(see Table 2; see supplements for results per wave).

**Table 2:** Descriptives of associated and outcome variables

Variables	*N* available	Descriptives total sample (*n* = 673)
*Outcome variables*		
Intention to use (M ± SD; range)	*N* = 630	3.1 ± 3 (1–5)
Perceived satisfaction (M ± SD; range)	*N* = 631	6.4 ± 2.4 (0–10)
*Associated variables*		
Mean age ± SD (range)	*N* = 647	60.6 ± 13.9 (18–94)
Gender (%)	*N* = 647	Female: 440(68%); Male: 205 (31.7%); Non-binary: 2 (0.3%)
Employment situation (%)	*N* = 643	Paid job: 260 (40,4%); Volunteering: 100 (15.6%); Unemployed: 249 (38.7%); I prefer not to tell: 4 (0.6%); Other, namely: 30 (4.7%)*
Educational attainment (%)	*N* = 643	Low: 168 (26.1%); Medium: 225 (35.0%); High: 250 (38.9%)
Ethnicity	*N* = 123**	Dutch: 96 (78.0%); Surinam: 8 (6.5%); Türkiye: 7 (5.7%); Other ***
Financial scarcity (M ± SD; range)	*N* = 639	9 ± 4.9 (4–28)
Health literacy (M ± SD; range)	*N* = 643	3.1 ± 0.6 (1–4)
General health perception	*N* = 507****	Excellent: 29 (5.7%); Very good: 108 (21.3%); Good: 240 (47,3%); Fair: 117(23,1%); Poor: 13 (2.6%)
Dementia risk (M ± SD; range)	*N* = 627	−2.1 ± 2.7 (−5.9-8.2)
MCLHB-DRR: perceived severity (M ± SD; range)	*N* = 628	2.9 ± 0.9 (1–5)
MCHLB-DRR: perceived benefits (M ± SD; range)	*N* = 628	2.7 ± 0.8 (1–5)
MCHLB-DRR: perceived barriers (M ± SD; range)	*N* = 628	3.8 ± 0.7 (1–5)
MCHLB-DRR: cues to action (M ± SD; range)	*N* = 628	3.1 ± 0.8 (1–5)
MCHLB-DRR: perceived susceptibility (M ± SD; range)	*N* = 628	3.2 ± 0.9 (1–5)
MCHLB-DRR: general health motivation (M ± SD; range)	*N* = 628	2 ± 0.9 (1–5)
MCHLB-DRR: self-efficacy (M ± SD; range)	*N* = 628	2.8 ± 0.9 (1–5)
Digital proficiency (M ± SD; range)	*N* = 628	36.1 ± 5.8 (8–40)
Availability of digital technology at home	*N* = 507****	Smartphone: 494 (97,4%); Laptop: 365 (72.0%); Computer: 195 (38.5%); Tablet: 320 (63.1%); Wearable: 158 (31.2%); Game computer: 52 (10.3%); Other: 11 (2.2%); I do not have or use digital tools at home: 1 (0.2%)
Digital acceptability (M ± SD; range)	*N* = 627	17.6 ± 3 (10–25)
Technology adoption (M ± SD; range)	*N* = 627	3 ± 0.4 (1.36–4.36)

Notes. Range refers to observed range in our dataset

*Other comprised for instance self-employed, sickness or unemployment benefits, or pre-retirement

**Only available for wave II

***Poland: 2 (1.6%); Morocco: 2 (1.6%); Germany: 2 (1.6%); Caribbean Netherlands: 2 (1.6%); Gambia: 1 (0.8%); Belgium: 1 (0.8%); Iran: 1 (0.8%); Czech Republic: 1 (0.8%)

****Only available for wave I. We found no significant difference in mean scores for intention to use (*p* = 0.628) and perceived satisfaction (*p* = 0.480) between participants from wave I and wave II (see metadata; 10.34894/Z74Q0Y)

The mean intention to use was 3.1 (SD = 3; range = 1–5) and mean perceived satisfaction was 6.4 (SD = 2.4; range = 0–10) (see Table 2).

Tables 3 and 4 display results of the robust stepwise backward regression using 5-fold cross-validation that we conducted to explain the variance in both intention to use and perceived satisfaction scores. Of intention to use, 44.4% of the variance was explained by educational attainment, digital proficiency, digital acceptability, technology adoption, and cues to action (Table 3; see metadata for individual fold results and alpha values; 10.34894/Z74Q0Y). This means that those with higher educational attainment, digital proficiency, digital acceptability, technology adoption, as well as those with lower cues to action had higher intention to use MijnBreincoach. Sub-group analyses did not show significant differences between the < 40 and ≥ 40 age groups and the < 60 and ≥ 60 age groups for intention to use MijnBreincoach (see Table 3).

**Table 3:** Robust backward Stepwise regression outcomes using 5-fold cross-validation for intention to use

Baseline model	Educational attainment, gender, age, digital proficiency, digital acceptability, technology adoption, and cues to action
Robust co-variables in 5-fold cross-validation	• Educational attainment (β= −0.043; lower educational attainment relative to higher educational attainment) • Digital proficiency (β = 0.017) • Digital acceptability (β = 0.041) • Technology adoption (β = 1.267) • Cues to action (β= −0.122)
Mean R-squared	44.4%
T-test 40- versus 40 + model	*P* = 0.101
T-test 60- versus 60 + model	*P* = 0.927

For perceived satisfaction, 28.2% of the variance was explained by digital proficiency and technology adoption (see Table 4, see metadata for individual fold results and alpha values; 10.34894/Z74Q0Y). This means that those with higher digital proficiency and technology adoption were more inclined to recommend MijnBreincoach to someone else with cognitive complaints. For perceived satisfaction, no difference was found between the < 40 and ≥ 40 age groups, yet we did find a significant difference for the < 60 and ≥ 60 age group (*p* = 0.05; see Table 4). When re-running the 5-fold cross validation on the < 60 age group for perceived satisfaction, we found that 43.6% of the variance could be explained by technology adoption, educational attainment, and perceived barriers (see metadata for individual fold results and alpha values; 10.34894/Z74Q0Y). This means that those with higher technology adoption, educational attainment, and lower perceived barriers were more inclined to recommend MijnBreincoach to someone else. For the ≥ 60 age group, 26.4% of the variance was explained by technology adoption, and digital proficiency, indicating that those with higher technology adoption and digital proficiency were more likely to recommend MijnBreincoach to someone else.

**Table 4:** Robust backward Stepwise regression outcomes using 5-fold cross-validation for perceived satisfaction

Baseline model	Gender, digital proficiency, digital acceptability, technology adoption, general health motivation, self-efficacy
Robust co-variables in 5-fold cross-validation	• Digital proficiency (β = 0.090) • Technology adoption (β = 2.563)
Mean R-squared	27.8%
T-test 40- versus 40 + model	*P* = 0.387
T-test 60- versus 60 + model	*P* = 0.047
Robust co-variables in 5-fold cross- validation for < 60 age group	• Technology adoption (β = 3.206) • Educational attainment (β = −0.222) • Perceived barriers (β = 0.094)*
Mean R-squared for the < 60 age group	43.6%
Robust co-variables in 5-fold cross-validation for ≥ 60 age group	• Technology adoption (β = 2.455) • Digital proficiency (β = 0.095)
Mean R-squared for the ≥60 age group	26.4%

*Rescored for analysis purposes. A positive score thus means lower perceived barriers

## Discussion

Our study shows that the Dutch general public on average has a moderate intention to use MijnBreincoach, a digital tool for dementia prevention. In addition, they would not strongly recommend this tool to others with concerns about their brain health, which reflects relatively low perceived satisfaction. However, much variation in intention to use and perceived satisfaction scores was observed and significant associated variables were identified. Higher intention to use MijnBreincoach was observed among those with higher digital proficiency, digital acceptability, technology adoption, and educational attainment, and those who perceived lower cues to action. In addition, those with higher digital proficiency and technology adoption were more inclined to recommend MijnBreincoach to someone else with cognitive complaints. A more in-depth analysis of the influence of age revealed that people older than 60 years had different associated variables (technology adoption and digital proficiency) affecting perceived satisfaction in terms of recommending the tool to others compared to people younger than 60 years (technology adoption, educational attainment, and perceived barriers), but not for intention to use.

Our finding that people in the Dutch general public on average have a moderate intention to use digital tools reflects room for improvement. This is in line with other studies finding a moderate intention to use mHealth among Dutch older adults (e.g. 50.3% having intention to use) [70]. Following the Net Promoter Score (NPS) classification, the observed mean satisfaction score of 6.4 suggests a qualification of our sample as ‘detractors’, meaning that these are (potential) users who are unsatisfied and who are unlikely to recommend the tool to others [49, 71]. Our findings suggests that at least a moderate part of the Dutch general public may be inclined to a use a dementia prevention tool such as MijnBreincoach, yet that it is less likely that they will actively promote the digital tool to others. The moderate enthusiasm may be due to the fact that MijnBreincoach does not contain a clear component on in-person counselling. For instance, a consultation with a lifestyle coach seems to work well for weight-loss and nutrition-related interventions [72, 73]. Previous research on group exercise interventions, depression, and cognitive behaviour therapy also shows that people appreciate a blended intervention element containing a combination of a digital tool with in-person support, for instance, because this offers more continuous support [74–77]. A blended approach in which MijnBreincoach is combined with online or offline in-person coaching is recommended as previous research found that a blended approach has beneficial effects on lifestyle improvements [16, 78, 79]. Another explanation may be that it is likely that the digital medium itself is not (entirely) suitable for a proportion of the Dutch general public and that one particular digital tool is unlikely to meet the needs of all members of the public. For instance, because needs differ among people and may change over time. These explanations together may explain the moderate intention to use and perceived satisfaction we found on a group level, even though we also observed much variation in scores, suggesting that a dementia prevention tool like MijnBreincoach would be the ‘right’ tool for some but not for all.

The observed variance in intention to use and perceived satisfaction was explained, at least in part, by various associated variables, as mentioned above. Our findings are in line with previous research showing that adequate digital proficiency is necessary to effectively select and use eHealth and mHealth tools [80, 81]. Intention to use is also a key factor in technology adoption [48, 63]. It is thus not surprising that digital proficiency and technology adoption were found to be robustly associated with both intention to use and perceived satisfaction in this study. We also found an association between digital acceptability and intention to use a digital health tool. Digital acceptability is a key component of hallmark behaviour change theories in health psychology, including the Technology Acceptance Model and Theory of Planned Behaviour [82]. Previous research has also shown that individuals with higher levels of educational attainment tend to have a greater intention to use eHealth compared to those with lower educational attainment [83–85]. Moreover, higher educational attainment is often linked to increased digital proficiency and eHealth literacy, both of which positively influence the intention to use eHealth [84, 86]. This aligns with our finding that higher educated participants were more inclined to use digital tools and, among people aged < 60, to recommend these tools to others. These results, combined with previous research, highlight the importance of taking differences in digital proficiency, technology adoption, digital acceptability, and educational attainment into account when designing digital tools, research, and policies aimed at dementia prevention, to ensure inclusive and sustainable implementation of (public) health innovations.

Unexpectedly, we did not find a robust association between health literacy and intention to use a digital tool for dementia prevention. In this study, we measured functional health literacy, digital proficiency, and digital acceptability. It may be that this combination did not capture potential underlying, yet relevant, concepts that could influence intention to use, such as eHealth literacy. Previous literature shows that higher levels of both health and eHealth literacy tend to be positively correlated with mHealth app use and health outcomes [87]. Perceived eHealth literacy was also found to be positively related to user satisfaction with regard to continued adoption [88]. In future studies, it is thus recommended to incorporate an eHealth literacy measure, such as the eHEALS scale, to get more insight on this matter [89]. 

Another unexpected finding was that only the cues to action subscale of the Motivation to Change Lifestyle and Health Behaviours for Dementia Risk Reduction scale (MCLHB-DRR) showed robust association with intention to use, with those perceiving more external triggers to change their behaviour reporting less inclination to use MijnBreincoach. This was unexpected because of two reasons: the other subscales not showing any robust associations, and the negative direction of the association. It is possible that individuals who perceive many cues to action may think that they already have a relatively healthy lifestyle and may thus report lower motivation to change their behaviour using a digital app. The mean LIBRA index of the study sample is relatively low, reflecting good (self-reported) brain health. MijnBreincoach, the use case in this study, increases awareness of someone’s personal dementia risk profile and identifies room-for-improvement. In this way, the tool addresses the precontemplation, contemplation, preparation, and action stage of the Transtheoretical Model and Stages of Change [90, 91]. Participants in this study who experience high cues to action may have already passed the (pre-)contemplation stage. This could explain why they have lower intentions to use, as it pertains to stages that have already been traversed, thus not adequately addressing their needs. The other subscales of the MCHLB-DRR did not show robust associations. This finding suggests that the MCLHB-DRR may exclusively capture general motivation for lifestyle change, rather than motivation for specific behaviours or lifestyle aspects. For instance, someone’s motivation for engaging in physical activity might be different compared to that person’s motivation for engaging in healthy eating or drinking less alcohol. Also, other instruments in this questionnaire may already explain variance due to overlap with subscales of the MCHLB-DRR. For instance, the technology adoption model also contains questions on facilitating conditions and self-efficacy, which could have made the MCHLB-DRR subscales on self-efficacy and perceived barriers redundant. One of the limitations of regression analysis is indeed that overlap between variables may be missed, also referred to as the ‘regression trap’ [92, 93]. 

Some previous research showed that people are less likely to be included in the digital world as they get older [94]. This may indicate that older adults have relatively less access to digital tools than younger adults or may be less willing to use these. However, in our study, age was not associated with intention to use or perceived satisfaction. In subgroup analyses, we found that people older than 60 years had different associated variables affecting perceived satisfaction in terms of recommending the tool to others compared to people younger than 60 years, but not for intention to use. This is in line with previous research on MijnBreincoach that also did not find any differences in app use between age groups [16]. Older adults are increasingly using telehealth, smartphone apps, and other digital health technologies, which explains why age might not have a strong influence [95]. Moreover, the potential difference between older and younger adults’ digital use is not necessarily about differences in actual digital skills, but could also be explained by their educational attainment, socioeconomic position, self-efficacy, and/or tendency to underestimate their digital skills [96–101]. According to some scholars, the misconception that older adults are less digitally skilled and are therefore perceived to be unwilling and unable to use digital tools can be seen as a form of ageism [102, 103]. Taken together, age in itself is unlikely to influence intention to use and perceived satisfaction of digital tools, but it is rather in combination with other factors.

Our findings did show that those younger than 60 years who perceived less barriers for changing one’s health behaviour for dementia risk reduction seem to show higher intention to use digital tools. Perceived barriers is a subscale of the MCHLB-DRR and comprises questions, for instance, addressing time, finances, and planning. Perceived barriers was not found to show a robust association for those younger than 40 or those 60 years and older, thus showing that the influence of perceived barriers on intention to use digital tools seems to specifically apply to people in midlife (40–60 years). People in midlife usually have limited time compared to other age groups due to ‘double’ responsibilities and other life circumstances, such as having a job and caring for children, parents, a partner, and/or others [104]. This would imply that digital tools aimed at people in midlife might benefit from taking perceived barriers into account during tool or intervention design and implementation.

This study has several strengths and limitations. The substantial sample size contributed to the reliability and validity of our findings. The following limitations also need acknowledgement. Firstly, in order to maximize study sample diversity as much as possible, we divided recruitment in two waves with special attention of enriching our existing dataset in wave II. For this purpose, we removed certain items from our questionnaire in wave II for questionnaire length reduction reasons, which may have led to different results from our sample in wave I and suboptimal comparisons between wave I and II for certain variables. Even though we tried our best to maximize the study sample diversity in both wave I and wave II, we still had limited study sample diversity regarding digital literacy, ethnicity, and financial scarcity, which negatively affects the generalizability of our results (see Kaijser et al., in preparation). Secondly, during recruitment, we informed people about the study topic, which may have resulted in people who are not keen on digital tools to not participate, thus, resulting in selection bias in our study sample. Thirdly, a use case measures the perception of use of a digital tool, and does not measure actual use and interaction with a digital tool, which results in the fact that we were only able to measure perceived satisfaction instead of actual satisfaction. Fourthly, the questionnaire in wave II included only one use case instead of four use cases in wave I (see supplements S2 and metadata; 10.34894/Z74Q0Y), and therefore, we only considered the use case in both wave I and wave II in our analysis, which could have influenced our results. We chose to use MijnBreincoach as the use case instead of the other use cases, because this tool had the highest mean behavioural intention score. Finally, backward stepwise regression is a form of multiple testing, which increases the chance on type I error. We could not control for multiple testing, for instance, with Bonferroni, as in stepwise regression one does not know beforehand how many tests one has to perform. As mentioned, regression analyses are also not able to capture overlap between variables [92, 93]. Our results are thus explicitly exploratory and should be interpreted with caution in light of this risk for type I error.

As a future research direction, qualitative follow-up research can gain a more in-depth understanding of people’s intention to use a specific tool and what contributes to their perceived satisfaction, including those who have limited literacy, speak no Dutch, or need a more extensive relationship and trust before participating in research. As this study is exploratory in nature, follow-up research to confirm our findings is recommended that controls for the limitations of a regression analysis and uses a measure to assess eHealth literacy. As for practical implications, there seem to be several potentially modifiable factors associated with intention to use and perceived satisfaction, which provide opportunities for the design, dissemination strategies, and public health policy of digital tools for dementia prevention. If people are expected to take a more active role in their own brain health by using digital tools, the usability, inclusivity, and accessibility of these tools should be guaranteed. From ethical, impact, and human-centred design perspective, it is important to focus on involving diverse end-users from the beginning in development and user testing, for instance, via co-design [105, 106]. Our study further emphasizes the importance of including people with varying educational attainment, digital proficiency, and digital acceptability in the design and development of digital tools for dementia prevention when it comes to diversity. Moreover, it is recommended to specify intended groups of end-users, and develop and offer a variety of digital tools tailored to different groups, as people differ in their needs and preferences. When looking at the context in which digital tools will be offered to the general public, digital proficiency and digital acceptability may be enhanced via creating (blended) support services, and policies on a local and national level (e.g. subsidies and availability of trainings and digital tools; keep offering alternatives if people are not willing or have the access to using digital tools) and inclusive development of digital tools.

## Conclusion

The Dutch general public has a moderate intention to use and perceived satisfaction with a digital tool for dementia prevention. Yet, large variation in scores was observed, which seem to be explained by differences in educational attainment, digital proficiency, and digital acceptability. A wide range of end-users with diverse backgrounds should thus be involved in digital tool design and development from the beginning. Digital dementia prevention tools should also be tailored to specific target populations with the goal of creating a diverse array of tools and services, including an offline alternative if people are not willing or have access to use digital tools. All with the ultimate aim to facilitate inclusive and sustainable evidence-based public health practices aimed at dementia prevention.

## Supplementary Information

Supplementary Material 1.

## Data Availability

The data that support the findings of this study are available upon reasonable request and permission by the Amsterdam UMC in a restricted access location at the Amsterdam UMC, location AMC. Metadata are published online in Dataverse and are accessible via: https://doi.org/10.34894/Z74Q0Y.

## Abbreviations

AD: Alzheimer’s disease
NPS: Net Promoter Score
UTAUT-2: Unified Theory of Acceptance & Use of Technology-2
PIFS-4: Psychological Inventory of Financial Scarcity-4
FCCHL: Functional, Communicative, and Critical Health Literacy
LIBRA: Lifestyle for Brain Health Scores
MCHLB-DRR: Motivation to Change Lifestyle and Health Behaviours for Dementia Risk Reduction scale
MDPQ-16: Mobile Device Proficiency Questionnaire
MoHTAM: mHealth Technology Acceptance Model
NPS: Net Promoter Score
GLM: Generalized linear models
