# Supplementary material for: Intention to use and perceived satisfaction with a digital tool for dementia prevention among the Dutch general public: a cross-sectional study
Source: BMC Public Health. 2025 Sep 30;25:3151. doi: 10.1186/s12889-025-23777-y (PMC12486987; doi:10.1186/s12889-025-23777-y)
Supplement: Supplementary file 1 — Supplementary Material 1. [file 12889_2025_23777_MOESM1_ESM.docx]

# Supplementary materials for manuscript: ‘Intention to use and perceived satisfaction with a digital tool for dementia prevention among the Dutch general public: a cross-sectional study’

## S1.Theoretical framework

The intention to use digital tools can be explained from a health behavior perspective. The I-Change Model (26) is a recent, frequently used model to explain health-related behavior and is based on key models from health psychology, such as the Theory of Planned Behavior, Social Cognitive Theory, and the Health Belief Model. The model states that behavior is mainly informed by behavioral intentions and abilities (26, 95), which can be subdivided into predisposing factors (biological, psychological, behavioral, and socio-cultural), awareness factors (knowledge, cue for action, and risk perception), information factors (message, channel, and source), and motivation factors (attitude, social influence, and self-efficacy). (26, 95) Behavioral intention (precontemplation, contemplation, and preparation) precedes actual behavior (perform and retain), and this behavior-intention gap is influenced by ability (implementation plans and physical skills) and barriers (thresholds)(26, 95) (see Figure 1).


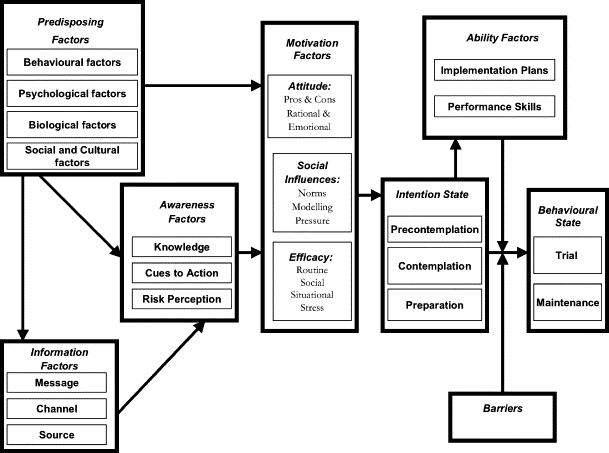


**Figure S1. The I-CHANGE model and figure by Hein de Vries (26, 95).**

The accessibility of digital tools may be understood by the Causal and Sequential Model of Digital Technology Access by Van Dijk. (27-33) It presupposes that existing inequalities among individuals lead to an unequal division of resources, which leads to unequal access to digital tools. (27) The model posits four successive kinds of access, including motivational access (people must be motivated to use digital tools), material access (people must have access to e.g. a smartphone and internet connection), skills access (people must have digital skills that are required to use the digital tool, e.g. swiping on a smartphone), and usage access (people must be able to use different apps and services, e.g. being able to download the tool from the Google Play or Apple store). (27) These successive kinds of access to digital tools are influenced by resources (e.g. material, mental or social), positional categories (e.g. sex, age, education or labor), participation in society (e.g. culture, economy, or social networks), and technological properties of the digital tool (e.g. hardware, software, or content) (see Figure 2). (96)


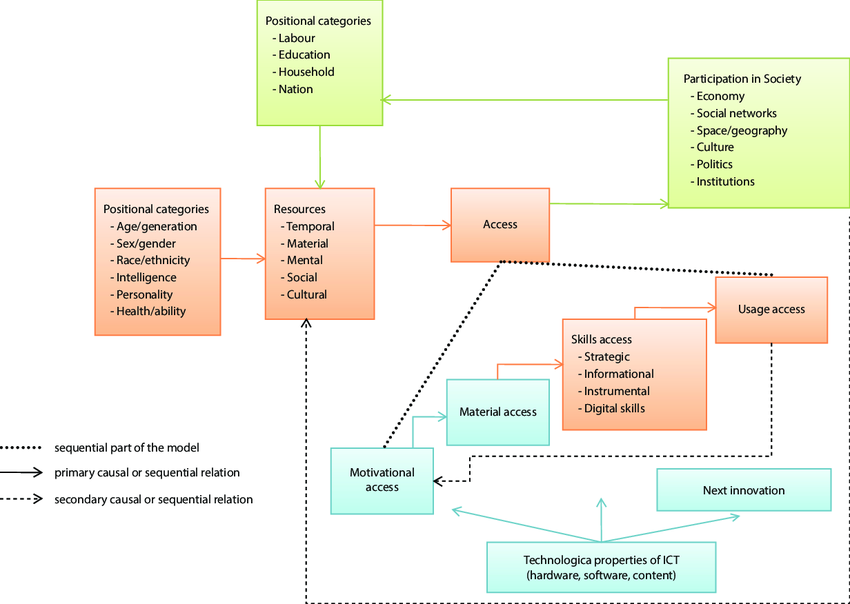


**Figure S2. The Causal and Sequential Model of Digital Technology Access (27-33). Figure is made by Nicole Goedhart (33).**

These two models partly overlap, e.g., ability versus skill access, or motivation factors versus motivational access to use digital tools. Taken together, these models have the potential to capture aspects associated with people’s intention to use and perceived satisfaction with digital tools for dementia prevention.

**S2. Difference in measures between wave I and wave II**

From wave I, we received feedback indicating that the questionnaire was relatively lengthy. We, therefore, shortened or removed certain questions and scales for wave II (see supplementary Table 5). It was crucial to take this feedback into account, since our aim for wave II was to enrich our study sample diversity, including for instance, people who have low literacy levels. After discussions and consensus in the team (TR, TE, ES, LV), we removed the use cases on three other types of digital tools from our questionnaire (cCog, ADappt, and Neurokeys) and focused on MijnBreincoach alone. For wave II, we chose for MijnBreincoach instead of the other tools, as this is a tool that exists and is a dementia prevention tool with our study sample as indented end-users. We also removed the question on general health perception and the availability of digital tools at home for pragmatic reasons. We shortened the question on employment situation by providing less answer options (‘*I have a paid job, I am volunteering, I am not employed, I prefer not to say, or other’*) and thus making the question less complicated. We also decided to only use the functional scale of the FCCHL questionnaire. Finally, we used a shortened version of the MCLHBDRR. The original version contains 27 items that reflect the subscales perceived susceptibility, perceived severity, perceived benefits, perceived barriers, cues to action, and general health information. (45) In Wave II, we used a shortened fourteen item version of the MCHLB-DRR that is shortened on subscale level based on statistics and theoretical grounds, which is described by Albers et al. (in preparation). Item-rest correlation was done to check if scales from wave I and wave II measure the same and no serious conflicts were found.

**Table S1. Overview of variables and instruments in wave I and wave II**

| Part | Variables | Instruments | Scoring | Cronbach’s alpha based on our sample |
| --- | --- | --- | --- | --- |
| *Associated variables* | | | | |
| Sociodemographics | Gender | - | - | - |
|  | Age in years | - | - | - |
|  | Employment situation | - | - | - |
|  | Educational attainment | Dutch CBS division by Pleijers & De Vries (55). | 1-item scale from 1 (low educational attainment) to 10 (high educational attainment) based on the Dutch educational system. Classification was done as follows: low (none or elementary school, primary or preparatory vocational education (vso, vmbo-b, vmbo-k, vmbo-g, vmbo-t, mavo, mulo, lts, leao, lhno of meao), lower grades of secondary school of HAVO or VWO, or assistant training (MBO-1)), medium (higher grades of secondary school of HAVO, VWO, HBS, or MMS, basic vocational training (MBO-2), vocational training (MBO-3), and middle management and specialist education (MBO-4)), and high (higher vocational education (HBO, HEAO, or HTS) and university). | - |
|  | Perceived financial scarcity | Psychological Inventory of Financial Scarcity (PIFS-4)(40, 41) | 4-item scale in which answers are given on a seven-point Likert scale ranging from strongly disagree (1 point) to strongly agree (7 points), so higher scores (min. 4; max. 28) reflect greater perceived experience financial scarcity (40). | α=0.796 |
|  | Ethnicity* | - | - | - |
| (Perceived) dementia risk and motivation for behaviour change | Health literacy** | Functional scale of the Functional, Communicative, and Critical Health Literacy (FCCHL) scale(42, 43) | Wave I: An eleven-item four-point Likert scale ranging from 1 (never) to four (often), so higher scores (min. 1; max. 4) reflect lower health literacy.  Wave II: A four-item four-point Likert scale ranging from 1 (never) to four (often), so higher scores (min. 1; max. 4) reflect lower health literacy. | α=0.700 |
|  | General health perception | - | - | - |
|  | Dementia risk | Lifestyle for Brain Health scores (LIBRA)(44) | Twelve items measuring LIBRA scores. Total scores range from -5.9 (min.) to 12.7 (max.), with a score above 1 being associated with higher scores reflecting a higher risk of dementia (and lower scores reflecting better brain health)(97, 98). | - |
|  | Motivation for dementia prevention-related behavior change** | Motivation to Change Lifestyle and Health Behaviours for Dementia Risk Reduction scale (MCLHB-DRR) (45, 99). | Wave I: A twenty-four-item five-point Likert scale. For analysis purposes, the Likert scale was recoded into 1 (low motivation) to 5 (motivation motivation).  (min. 1; max. 5).  Wave II: A fourteen-item five-point Likert scale. For analysis purposes, the Likert scale was recoded into 1 (low motivation) to 5 (motivation motivation).  (min. 1; max. 5). | Perceived susceptibility: N/A*****; perceived severity: α=0.659; perceived benefits: α=0.390; perceived barriers: α=0.673; cues to action: α=0.692; general health motivation: N/A*****; self-efficacy: N/A***** |
| Digital proficiency and acceptability | Digital proficiency | Mobile Device Proficiency Questionnaire (MDPQ-16)(46) | A sixteen-item five-point Likert scale is used with 1 being ‘never tried’ and 5 being ‘very easy’, so that higher scores reflect a higher mobile device proficiency (min. 8; max. 40). | α=0.927 |
|  | Availability of digital technology at home* | - | - | - |
|  | Digital acceptability | Trust, perceived risk, and resistance to change items of mHealth Technology Acceptance Model (MoHTAM)(47) | Six questions are asked using a five-point Likert scale, with 1 being ‘completely agree’ and 5 being ‘strongly disagree’. A lower score means lower trust, perceived risk, and acceptance of technology (min. 6; max. 30). | - |
| Use case assessing behavioral intentions and perception of usability | Technology adoption**** | Unified Theory of Acceptance & Use of Technology 2 (UTAUT-2) (38) | A fourteen-item five-point Likert scale is used with 1 being ‘strongly agree’ and 5 being ‘strongly disagree’. For analysis purposes, the Likert scale was recoded into 1 being ‘strongly disagree’ and 5 being ‘strongly agree’, so that higher total scores indicate a higher intention (min. 1; max. 5). | α=0.883**** |
| *Outcome variables* | | | | |
| Use case assessing behavioral intentions and perception of usability | Intention to use | Unified Theory of Acceptance & Use of Technology 2 (UTAUT-2) (38) | A three-item five-point Likert scale is used with 1 being ‘strongly agree’ and 5 being ‘strongly disagree’. For analysis purposes, the Likert scale was recoded into 1 being ‘strongly disagree’ and 5 being ‘strongly agree’, so that higher total scores indicate a higher intention (min. 1; max. 5). | - |
|  | Perceived satisfaction | Net Promoter Score (NPS) | The NPS is a one-item question measured on a scale of 0 being ‘not at all likely’ and 10 being ‘very likely’(39). | - |

*Notes. *only available for wave II. *** *wave I: the entire scale; wave II: shortened version. ***only available for wave I. ****excluding ‘intention to use’ items. *****N/A, since subscale consists of one item.*

**S3. Demographics of wave I, wave II, and both waves combined**

**Table S2. Demographics**

| Factors and covariates | Descriptives total sample (n=673) | Descriptives sample wave I (n=507) | Descriptives wave II (n=166) |
| --- | --- | --- | --- |
| *Associated variables* | | | |
| Mean age (mean±SD; range; n) | 60.6±13.9 (18-94) (N=647) | 60.6±14.3 (20-89) (N=507) | 60.7±12.7(18-94) (N=140) |
| Gender (%) | Female: 440/647 (68%)  Male: 205/647 (31.7%)  Non-binary: 2/647 (0.3%) | Female: 349/507 (68.8%) Male: 158/507 (31.2%) | Female: 91/140 (65.0%)  Male: 47/140 (33.6%) Non-binary: 2/140 (1.4%) |
| Employment situation (%) | Paid job: 260/643 (40,4%)  Volunteering: 100/643 (15.6%)  Unemployed: 249/643 (38.7%)  I prefer not to tell: 4/643 (0.6%)  Other, namely: 30/643 (4.7%) | Paid job: 199/507 (39.3%)  Volunteering: 89/507 (17.6%)  Unemployed: 218/507 (43.0%)  I prefer not to tell: 1/507 (0.2%) | Paid job: 61/136 (44.9%)  Volunteering: 11/136 (8.1%)  Unemployed: 31/136 (22.8%)  I prefer not to tell: 3/136 (2.2%)  Other, namely: 30/136 (22.1%) |
| Educational attainment (%) | Low: 168/643 (26.1%)  Medium: 225/643 (35.0%)  High: 250/643 (38.9%) | Low: 132/507 (26.0%)  Medium: 184/507 (36.3%)  High: 191/507 (37.7%) | Low: 36/136 (26.5%)  Medium: 41/136 (30.1%)  High: 59/136 (43.4%) |
| Ethnicity* | Dutch: 96/123 (78.0%)  Surinam: 8/123 (6.5%)  Türkiye: 7/123 (5.7%)  Poland: 2/123 (1.6%)  Morocco: 2/123 (1.6%)  Germany: 2/123 (1.6%)  Caribbean Netherlands: 2/123 (1.6%)  Gambia: 1/123 (0.8%)  Belgium: 1/123 (0.8%)  Iran: 1/123 (0.8%)  Czech Republic: 1/123 (0.8%) | - | Dutch: 96/123 (78.0%)  Surinam: 8/123 (6.5%)  Türkiye: 7/123 (5.7%)  Poland: 2/123 (1.6%)  Morocco: 2/123 (1.6%)  Germany: 2/123 (1.6%)  Caribbean Netherlands: 2/123 (1.6%)  Gambia: 1/123 (0.8%)  Belgium: 1/123 (0.8%)  Iran: 1/123 (0.8%)  Czech Republic: 1/123 (0.8%) |
| Financial scarcity (mean±SD; range; N) | 9±4.9 (4-28) (N=639) | 8.6±4.5 (4-24) (N=505) | 10.6±5.9 (4-28) (N=134) |
| Health literacy  (mean±SD; range;N) | 3.1±0.6 (1-4) (N=643) | 1.9±0.5 (1-3.5) (N=507) | 2.1±0.8 (1-4) (N=136) |
| General health perception** | Excellent: 29/507 (5.7%)  Very good: 108/507(21.3%)  Good: 240/507 (47,3%)  Fair: 117/507(23,1%)  Poor: 13/507 (2.6%) | Excellent: 29/507 (5.7%)  Very good: 108/507(21.3%)  Good: 240/507 (47,3%)  Fair: 117/507(23,1%)  Poor: 13/507 (2.6%) | - |
| Dementia risk  (mean±SD; range; N) | -2.1±2.7 (-5.9-8.2) (N=627) | -2.1±2.6 (-5.9-8.2) (N=506) | -1.7±2.9 (-5.9-6.9) (N=121) |
| MCLHB-DRR: perceived severity  (mean±SD; range; N) | 2.9±0.9 (1-5) (N=628) | 2.9±0.9 (1-5)  (N=507) | 3±0.8 (1-5) (N=121) |
| MCHLB-DRR: perceived benefits  (mean±SD; range; N) | 2.7±0.8 (1-5) (N=628) | 2.6±0.8 (1-5)  (N=507) | 2.9±0.6 (1-4.5) (N=121) |
| MCHLB-DRR: perceived barriers  (mean±SD; range; N) | 3.8±0.7 (1-5) (N=628) | 3.9±0.7 (1-5)  (N=507) | 3.6±0.9 (1-5) (N=121) |
| MCHLB-DRR: cues to action  (mean±SD; range; N) | 3.1±0.8 (1-5) (N=628) | 3±0.8 (1-5)  (N=507) | 3.2±0.8 (1-5) (N=121) |
| MCHLB-DRR: perceived susceptibility  (mean±SD; range; N) | 3.2±0.9 (1-5) (N=628) | 3.2±0.9 (1-5)  (N=507) | 3.3±0.8 (1-5) (N=121) |
| MCHLB-DRR: general health motivation  (mean±SD; range; N) | 2±0.9 (1-5) (N=628) | 2±0.8 (1-5)  (N=507) | 2.1±1.1 (1-5) (N=121) |
| MCHLB-DRR: self-efficacy  (mean±SD; range; N) | 2.8±0.9 (1-5) (N=628) | 2.8±0.9 (1-5)  (N=507) | 2.8±0.9 (1-5) (N=121) |
| Digital proficiency  (mean±SD; range; N) | 36.1±5.8 (8-40) (N=628) | 36.5±5.2 (8-40) (N=507) | 34.3±7.6 (8-40) (N=121) |
| Availability of digital technology at home** | Smartphone: 494/507 (97,4%)  Laptop: 365/507 (72.0%)  Computer: 195/507 (38.5%)  Tablet: 320/507 (63.1%)  Wearable: 158/507 (31.2%)  Game computer: 52/507(10.3%)  Other: 11/507 (2.2%)  I do not have or use digital tools at home: 1/507 (0.2%) | Smartphone: 494/507 (97,4%)  Laptop: 365/507 (72.0%)  Computer: 195/507 (38.5%)  Tablet: 320/507 (63.1%)  Wearable: 158/507 (31.2%)  Game computer: 52/507(10.3%)  Other: 11/507 (2.2%)  I do not have or use digital tools at home: 1/507 (0.2%) |  |
| Digital acceptability  (mean±SD; range; N) | 17.6±3 (10-25) (N=627) | 17.6 ±3.1 (10-25) (N=507) | 17.6±2.6 (10-24) (N=120) |
| Technology adoption  (mean±SD; range; N) | 3±0.4 (1.36-4.36) (N=627) | 3±0.4 (1.4-4.4) (N=507) | 2.9±0.5 (1.6-4.4) (N=120) |
| *Outcome variables* | | | |
| Intention to use  (mean±SD; range; N) | 3.1±3 (1-5) (N=630) | MijnBreincoach tool: 2.88± 0.95 (1-5); cCog tool: 2.7±0.83 (1-5); ADappt tool: 2.77±0.9(1-5); Neurokeys tool: 3.15±1.02 (1-5)  (N=507)**** | 3.2±0.9 (1-5) (N=123) |
| Perceived satisfaction  (mean±SD; range; N) | 6.4±2.4 (0-10) (N=631) | MijnBreincoach tool: =6.38± 2.44(0-10); cCog tool: 6.43±2.21 (0-10); ADappt tool: 6.42±2.26 (0-10); Neurokeys tool: 5.35±2.63 (0-10)  (N=507)**** | 6.2±2.3 (0-10) (N=124) |

*Notes.*data on ethnicity is only available for recruitment wave II.*

***Data on availability of technology at home is only available for recruitment wave I.
****In wave I, four different use cases were used, whereas in wave II, only the use case of MijnBreincoach was used.*

**S4. Correlation tables**

## Table S3. Explorative Pearson correlation matrix on intention to use.

|  |  | Intention to use (UTAUT) | Age in years | Health literacy (FCCHL) | Perceived susceptibility (MCHLB-DRR) | General Health motivation (MCHLB-DRR) | Self-efficacy (MCHLB-DRR) | Perceived severity (MCHLB-DRR) | Perceived benefits (MCHLB-DRR) | Perceived barriers (MCHLB-DRR) | Cues to action (MCHLB-DRR) | Mobile technology acceptance (MoHTAM) | Technology adoption (UTAUT-2) |
| --- | --- | --- | --- | --- | --- | --- | --- | --- | --- | --- | --- | --- | --- |
| Intention to use (UTAUT) | Pearson Correlation | 1 | ,017 | ,085^*^ | -,063 | -,122^*^ | -,189^*^ | -,149^*^ | -,161^*^ | ,016 | -,262^**^ | ,200^**^ | ,635^**^ |
|  | Sig. (2-tailed) |  | ,674 | ,032 | ,114 | ,002 | <,001 | <,001 | <,001 | ,681 | <,001 | <,001 | <,001 |
|  | N | 630 | 630 | 630 | 627 | 627 | 627 | 627 | 627 | 627 | 627 | 627 | 627 |
| Age in years | Pearson Correlation | ,017 | 1 | ,169^*^ | -,011 | -,075 | ,028 | -,102^*^ | ,073 | -,011 | -,064 | -,093^*^ | -,078 |
|  | Sig. (2-tailed) | ,674 |  | <,001 | ,776 | ,062 | ,486 | ,010 | ,068 | ,783 | ,111 | ,020 | ,052 |
|  | N | 630 | 647 | 643 | 628 | 628 | 628 | 628 | 628 | 628 | 628 | 627 | 627 |
| Health literacy (FCCHL) | Pearson Correlation | ,085^*^ | ,169^*^ | 1 | -,153^*^ | -,073 | ,062 | -,117^*^ | ,033 | -,204^**^ | -,156^**^ | -,123^**^ | ,094^*^ |
|  | Sig. (2-tailed) | ,032 | <,001 |  | <,001 | ,069 | ,123 | ,003 | ,412 | <,001 | <,001 | ,002 | ,019 |
|  | N | 630 | 643 | 643 | 628 | 628 | 628 | 628 | 628 | 628 | 628 | 627 | 627 |
| Perceived susceptibility (MCHLB-DRR) | Pearson Correlation | -,063 | -,011 | -,153^*^ | 1 | ,038 | -,049 | ,317^*^ | ,110^*^ | ,024 | ,278^**^ | ,022 | -,044 |
|  | Sig. (2-tailed) | ,114 | ,776 | <,001 |  | ,345 | ,224 | <,001 | ,006 | ,555 | <,001 | ,582 | ,267 |
|  | N | 627 | 628 | 628 | 628 | 628 | 628 | 628 | 628 | 628 | 628 | 627 | 627 |
| General Health motivation (MCHLB-DRR) | Pearson Correlation | -,122^*^ | -,075 | -,073 | ,038 | 1 | ,126^*^ | ,193^*^ | ,068 | -,184^**^ | ,119^**^ | -,045 | -,167^**^ |
|  | Sig. (2-tailed) | ,002 | ,062 | ,069 | ,345 |  | ,002 | <,001 | ,087 | <,001 | ,003 | ,257 | <,001 |
|  | N | 627 | 628 | 628 | 628 | 628 | 628 | 628 | 628 | 628 | 628 | 627 | 627 |
| Self-efficacy (MCHLB-DRR) | Pearson Correlation | -,189^*^ | ,028 | ,062 | -,049 | ,126^*^ | 1 | ,018 | ,441^*^ | -,109^**^ | ,314^**^ | -,064 | -,268^**^ |
|  | Sig. (2-tailed) | <,001 | ,486 | ,123 | ,224 | ,002 |  | ,655 | <,001 | ,006 | <,001 | ,108 | <,001 |
|  | N | 627 | 628 | 628 | 628 | 628 | 628 | 628 | 628 | 628 | 628 | 627 | 627 |
| Perceived severity (MCHLB-DRR) | Pearson Correlation | -,149^*^ | -,102^*^ | -,117^*^ | ,317^*^ | ,193^*^ | ,018 | 1 | ,142^*^ | ,110^**^ | ,326^**^ | ,002 | -,144^**^ |
|  | Sig. (2-tailed) | <,001 | ,010 | ,003 | <,001 | <,001 | ,655 |  | <,001 | ,006 | <,001 | ,957 | <,001 |
|  | N | 627 | 628 | 628 | 628 | 628 | 628 | 628 | 628 | 628 | 628 | 627 | 627 |
| Perceived benefits (MCHLB-DRR) | Pearson Correlation | -,161^*^ | ,073 | ,033 | ,110^*^ | ,068 | ,441^*^ | ,142^*^ | 1 | ,086^*^ | ,496^**^ | -,051 | -,224^**^ |
|  | Sig. (2-tailed) | <,001 | ,068 | ,412 | ,006 | ,087 | <,001 | <,001 |  | ,030 | <,001 | ,206 | <,001 |
|  | N | 627 | 628 | 628 | 628 | 628 | 628 | 628 | 628 | 628 | 628 | 627 | 627 |
| Perceived barriers (MCHLB-DRR) | Pearson Correlation | ,016 | -,011 | -,204^*^ | ,024 | -,184^*^ | -,109^*^ | ,110^*^ | ,086^*^ | 1 | ,148^**^ | ,128^**^ | ,052 |
|  | Sig. (2-tailed) | ,681 | ,783 | <,001 | ,555 | <,001 | ,006 | ,006 | ,030 |  | <,001 | ,001 | ,194 |
|  | N | 627 | 628 | 628 | 628 | 628 | 628 | 628 | 628 | 628 | 628 | 627 | 627 |
| Cues to action (MCHLB-DRR) | Pearson Correlation | -,262^*^ | -,064 | -,156^*^ | ,278^*^ | ,119^*^ | ,314^*^ | ,326^*^ | ,496^*^ | ,148^**^ | 1 | ,015 | -,275^**^ |
|  | Sig. (2-tailed) | <,001 | ,111 | <,001 | <,001 | ,003 | <,001 | <,001 | <,001 | <,001 |  | ,707 | <,001 |
|  | N | 627 | 628 | 628 | 628 | 628 | 628 | 628 | 628 | 628 | 628 | 627 | 627 |
| Mobile technology acceptance (MoHTAM) | Pearson Correlation | ,200^*^ | -,093^*^ | -,123^*^ | ,022 | -,045 | -,064 | ,002 | -,051 | ,128^**^ | ,015 | 1 | ,116^**^ |
|  | Sig. (2-tailed) | <,001 | ,020 | ,002 | ,582 | ,257 | ,108 | ,957 | ,206 | ,001 | ,707 |  | ,004 |
|  | N | 627 | 627 | 627 | 627 | 627 | 627 | 627 | 627 | 627 | 627 | 627 | 627 |
| Technology adoption (UTAUT-2) | Pearson Correlation | ,635^*^ | -,078 | ,094^*^ | -,044 | -,167^*^ | -,268^*^ | -,144^**^ | -,224^*^ | ,052 | -,275^**^ | ,116^**^ | 1 |
|  | Sig. (2-tailed) | <,001 | ,052 | ,019 | ,267 | <,001 | <,001 | <,001 | <,001 | ,194 | <,001 | ,004 |  |
|  | N | 627 | 627 | 627 | 627 | 627 | 627 | 627 | 627 | 627 | 627 | 627 | 627 |

*Notes. *Correlation is significant at the 0.05 level (2-tailed).*

**Table S4. Explorative Pearson correlation matrix on perceived satisfaction (NPS).**

|  |  | Age in years | Health literacy (FCCHL) | Perceived susceptibility (MCHLB-DRR) | General Health motivation (MCHLB-DRR) | Self-efficacy (MCHLB-DRR) | Perceived severity (MCHLB-DRR) | Perceived benefits (MCHLB-DRR) | Perceived barriers (MCHLB-DRR) | Cues to action (MCHLB-DRR) | Mobile technology acceptance (MoHTAM) | Technology adoption (UTAUT) | Perceived satisfaction (NPS) |
| --- | --- | --- | --- | --- | --- | --- | --- | --- | --- | --- | --- | --- | --- |
| Age in years | Pearson Correlation | 1 | ,169^**^ | -,011 | -,075 | ,028 | -,102^*^ | ,073 | -,011 | -,064 | -,093^*^ | -,078 | -,098^*^ |
|  | Sig. (2-tailed) |  | <,001 | ,776 | ,062 | ,486 | ,010 | ,068 | ,783 | ,111 | ,020 | ,052 | ,014 |
|  | N | 647 | 643 | 628 | 628 | 628 | 628 | 628 | 628 | 628 | 627 | 627 | 631 |
| Health literacy (FCCHL) | Pearson Correlation | ,169^*^ | 1 | -,153^*^ | -,073 | ,062 | -,117^*^ | ,033 | -,204^*^ | -,156^*^ | -,123^*^ | ,094^*^ | -,072 |
|  | Sig. (2-tailed) | <,001 |  | <,001 | ,069 | ,123 | ,003 | ,412 | <,001 | <,001 | ,002 | ,019 | ,069 |
|  | N | 643 | 643 | 628 | 628 | 628 | 628 | 628 | 628 | 628 | 627 | 627 | 631 |
| Perceived susceptibility (MCHLB-DRR) | Pearson Correlation | -,011 | -,153^*^ | 1 | ,038 | -,049 | ,317^*^ | ,110^*^ | ,024 | ,278^*^ | ,022 | -,044 | ,049 |
|  | Sig. (2-tailed) | ,776 | <,001 |  | ,345 | ,224 | <,001 | ,006 | ,555 | <,001 | ,582 | ,267 | ,224 |
|  | N | 628 | 628 | 628 | 628 | 628 | 628 | 628 | 628 | 628 | 627 | 627 | 628 |
| General Health motivation (MCHLB-DRR) | Pearson Correlation | -,075 | -,073 | ,038 | 1 | ,126^*^ | ,193^*^ | ,068 | -,184^*^ | ,119^*^ | -,045 | -,167^*^ | -,182^*^ |
|  | Sig. (2-tailed) | ,062 | ,069 | ,345 |  | ,002 | <,001 | ,087 | <,001 | ,003 | ,257 | <,001 | <,001 |
|  | N | 628 | 628 | 628 | 628 | 628 | 628 | 628 | 628 | 628 | 627 | 627 | 628 |
| Self-efficacy (MCHLB-DRR) | Pearson Correlation | ,028 | ,062 | -,049 | ,126^*^ | 1 | ,018 | ,441^*^ | -,109^*^ | ,314^*^ | -,064 | -,268^*^ | -,228^*^ |
|  | Sig. (2-tailed) | ,486 | ,123 | ,224 | ,002 |  | ,655 | <,001 | ,006 | <,001 | ,108 | <,001 | <,001 |
|  | N | 628 | 628 | 628 | 628 | 628 | 628 | 628 | 628 | 628 | 627 | 627 | 628 |
| Perceived severity (MCHLB-DRR) | Pearson Correlation | -,102^*^ | -,117^**^ | ,317^*^ | ,193^*^ | ,018 | 1 | ,142^**^ | ,110^*^ | ,326^*^ | ,002 | -,144^*^ | -,082^*^ |
|  | Sig. (2-tailed) | ,010 | ,003 | <,001 | <,001 | ,655 |  | <,001 | ,006 | <,001 | ,957 | <,001 | ,041 |
|  | N | 628 | 628 | 628 | 628 | 628 | 628 | 628 | 628 | 628 | 627 | 627 | 628 |
| Perceived benefits (MCHLB-DRR) | Pearson Correlation | ,073 | ,033 | ,110^*^ | ,068 | ,441^*^ | ,142^*^ | 1 | ,086^*^ | ,496^*^ | -,051 | -,224^*^ | -,135^*^ |
|  | Sig. (2-tailed) | ,068 | ,412 | ,006 | ,087 | <,001 | <,001 |  | ,030 | <,001 | ,206 | <,001 | <,001 |
|  | N | 628 | 628 | 628 | 628 | 628 | 628 | 628 | 628 | 628 | 627 | 627 | 628 |
| Perceived barriers (MCHLB-DRR) | Pearson Correlation | -,011 | -,204^*^ | ,024 | -,184^*^ | -,109^*^ | ,110^*^ | ,086^*^ | 1 | ,148^*^ | ,128^*^ | ,052 | ,137^*^ |
|  | Sig. (2-tailed) | ,783 | <,001 | ,555 | <,001 | ,006 | ,006 | ,030 |  | <,001 | ,001 | ,194 | <,001 |
|  | N | 628 | 628 | 628 | 628 | 628 | 628 | 628 | 628 | 628 | 627 | 627 | 628 |
| Cues to action (MCHLB-DRR) | Pearson Correlation | -,064 | -,156^*^ | ,278^*^ | ,119^*^ | ,314^*^ | ,326^*^ | ,496^*^ | ,148^*^ | 1 | ,015 | -,275^*^ | -,133^*^ |
|  | Sig. (2-tailed) | ,111 | <,001 | <,001 | ,003 | <,001 | <,001 | <,001 | <,001 |  | ,707 | <,001 | <,001 |
|  | N | 628 | 628 | 628 | 628 | 628 | 628 | 628 | 628 | 628 | 627 | 627 | 628 |
| Mobile technology acceptance (MoHTAM) | Pearson Correlation | -,093^*^ | -,123^*^ | ,022 | -,045 | -,064 | ,002 | -,051 | ,128^*^ | ,015 | 1 | ,116^*^ | ,179^*^ |
|  | Sig. (2-tailed) | ,020 | ,002 | ,582 | ,257 | ,108 | ,957 | ,206 | ,001 | ,707 |  | ,004 | <,001 |
|  | N | 627 | 627 | 627 | 627 | 627 | 627 | 627 | 627 | 627 | 627 | 627 | 627 |
| Technology adoption (UTAUT-2) | Pearson Correlation | -,078 | ,094^*^ | -,044 | -,167^*^ | -,268^*^ | -,144^*^ | -,224^*^ | ,052 | -,275^*^ | ,116^*^ | 1 | ,483^*^ |
|  | Sig. (2-tailed) | ,052 | ,019 | ,267 | <,001 | <,001 | <,001 | <,001 | ,194 | <,001 | ,004 |  | <,001 |
|  | N | 627 | 627 | 627 | 627 | 627 | 627 | 627 | 627 | 627 | 627 | 627 | 627 |
| Satisfaction (NPS) | Pearson Correlation | -,098^*^ | -,072 | ,049 | -,182^*^ | -,228^*^ | -,082^*^ | -,135^*^ | ,137^*^ | -,133^*^ | ,179^*^ | ,483^*^ | 1 |
|  | Sig. (2-tailed) | ,014 | ,069 | ,224 | <,001 | <,001 | ,041 | <,001 | <,001 | <,001 | <,001 | <,001 |  |
|  | N | 631 | 631 | 628 | 628 | 628 | 628 | 628 | 628 | 628 | 627 | 627 | 631 |

*Notes. *Correlation is significant at the 0.05 level (2-tailed).*

**Table S5. Explorative Spearman correlation matrix on intention to use.**

|  |  | Financial scarcity (PIFS-4) | Mobile device proficiency (MDPQ-16) | Dementia risk (LIBRA) | Intention to use (UTAUT-2) |
| --- | --- | --- | --- | --- | --- |
| Financial scarcity (PIFS-4) | Correlation Coefficient | 1,000 | -,127^*^ | ,216^*^ | ,020 |
|  | Sig. (2-tailed) | . | ,002 | <,001 | ,617 |
|  | N | 639 | 626 | 625 | 628 |
| Mobile device proficiency (MDPQ-16) | Correlation Coefficient | -,127^*^ | 1,000 | -,180^*^ | ,137^*^ |
|  | Sig. (2-tailed) | ,002 | . | <,001 | <,001 |
|  | N | 626 | 628 | 626 | 627 |
| Dementia risk (LIBRA) | Correlation Coefficient | ,216^*^ | -,180^*^ | 1,000 | ,014 |
|  | Sig. (2-tailed) | <,001 | <,001 | . | ,731 |
|  | N | 625 | 626 | 627 | 626 |
| Intention to use (UTAUT-2) | Correlation Coefficient | ,020 | ,137^*^ | ,014 | 1,000 |
|  | Sig. (2-tailed) | ,617 | <,001 | ,731 | . |
|  | N | 628 | 627 | 626 | 630 |

*Notes. *Correlation is significant at the 0.05 level (2-tailed).*

**Table S6. Explorative Spearman correlation matrix on perceived satisfaction (NPS).**

|  |  | Financial scarcity (PIFS-4) | Mobile device proficiency (MDPQ-16) | Dementia risk (LIBRA) | Perceived satisfaction (NPS) |
| --- | --- | --- | --- | --- | --- |
| Financial scarcity (PIFS) | Correlation Coefficient | 1,000 | -,127^*^ | ,216^*^ | -,016 |
|  | Sig. (2-tailed) | . | ,002 | <,001 | ,684 |
|  | N | 639 | 626 | 625 | 629 |
| Mobile device proficiency (MDPQ) | Correlation Coefficient | -,127^*^ | 1,000 | -,180^*^ | ,241^*^ |
|  | Sig. (2-tailed) | ,002 | . | <,001 | <,001 |
|  | N | 626 | 628 | 626 | 628 |
| Dementia risk (LIBRA) | Correlation Coefficient | ,216^*^ | -,180^*^ | 1,000 | -,066 |
|  | Sig. (2-tailed) | <,001 | <,001 | . | ,099 |
|  | N | 625 | 626 | 627 | 627 |
| Perceived satisfaction (NPS) | Correlation Coefficient | -,016 | ,241^*^ | -,066 | 1,000 |
|  | Sig. (2-tailed) | ,684 | <,001 | ,099 | . |
|  | N | 629 | 628 | 627 | 631 |

*Notes. *Correlation is significant at the 0.05 level (2-tailed).*
